# A Unique Case of a Malignant Sertoli Cell Tumour with Cutaneous Metastasis

**DOI:** 10.1100/tsw.2008.26

**Published:** 2008-02-06

**Authors:** C. Blick, S. Abdelhadi, D. Bailey, J. Kelleher, A. Muneer

**Affiliations:** ^1^ Harold Hopkins Department of Urology, Royal Berkshire Hospital, Reading, UK; ^2^ Department of Urology, Wycombe General Hospital, Buckinghamshire NHS Trust, UK

**Keywords:** Sertoli cell, malignant, metastatic

## Abstract

Pure Sertoli cell tumours (SCTs) represent less than 1% of testicular neoplasms and malignant forms are rare. We present a unique case of a 69-year-old man who initially underwent inguinal orchidectomy for a malignant SCT. He then subsequently developed a paraumbilical cutaneous lesion which was histologically identical to the primary tumour. SCTs rarely metastasise. This is the first case of SCT with cutaneous metastasis described in the literature.

## IMPORTANT MESSAGE

Osseous and lung metastasis via haematogenous spread are documented; however, cutaneous spread has not previously been described. Cisplatin-based chemotherapy has produced modest results in this situation[1]; however, the suggestion of a reduction in disease progression, although not quantified in this case, suggests that combination chemotherapy is an option in end-stage disease.

## CASE REPORT

A 69-year-old man was referred with a 3-month history of an enlarging right-sided scrotal swelling. He had previously undergone a vasectomy and a subsequent ultrasound scan showed a 1-cm well-circumscribed lesion consistent with a sperm granuloma. He was reassured and discharged. He re-presented 12 months later complaining of a progressive increase in size of the lesion. A repeat ultrasound scan now demonstrated a 2-cm hypoechoic lesion contiguous with the epididymis. The patient underwent an inguinal orchidectomy. The histology reported a poorly differentiated tumour composed of pleomorphic epitheliod cells. There is a high nuclear to cytoplasmic ratio with widespread necrosis and fibrosis, and numerous mitotic figures. This was associated with tumour extension into the spermatic cord. The tumour extended beyond the testis with lymphovascular invasion. Immunochemistry was positive for vimentin, MNF116, NSE, and EMA. CD30 was positive. As a result of the necrosis, lymphovascular invasion, infiltrative nature of the tumour with spread beyond the testis, and high mitotic rate, this tumour was diagnosed as a malignant Sertoli cell tumour (SCT) with both vascular and spermatic cord invasion. A staging CT scan revealed pulmonary and paraaortic metastases. At a subsequent oncology review, a periumbilical lesion was noted. This measured 100 × 80 mm, and was erythematous and nodular. The lesion was excised and the histology confirmed that it was a metastatic malignant SCT, measuring 25 × 25 × 40 mm, infiltrating the dermis and subcutaneous fat, showing vimentin positivity and negative for CEA and TTF-1, in keeping with the original scrotal lesion. The patient then received palliative chemotherapy consisting of etoposide and cisplatin, which appeared on repeat CT imaging to slightly slow the disease progression. The para-aortic and chest lesions remain, and at 2 years since the swelling was first noticed and 12 months postorchidectomy, this man died from disseminated disease.

Sertoli cells are epithelial, derived from the specific gonadal stroma which provide structural support to spermatogenic cells of the testis. Tumours involving these cells are classed as sex cord-stromal tumours which as a group account for 5% of all testicular tumours[1,2]. Most SCTs will have a benign clinical course, however, 10–20% of these will behave aggressively. Metastatic malignant SCTs are rare and carry a poor prognosis due to early metastatic disease[3]. Malignant presentation is usually a unilateral testicular swelling with or without gynaecomastia and the majority arise in normal intrascrotal testes. The lesion is most commonly solitary and unifocal, unlike benign lesions which tend to be bilateral and multifocal[3].There are no known genetic predispositions, but elevated oestrogen levels have been detected in some cases[4]. Metastatic spread has been described involving iliac, para-aortic, mediastinal, and peritoneal lymph nodes[3,4,5,6,7,8].

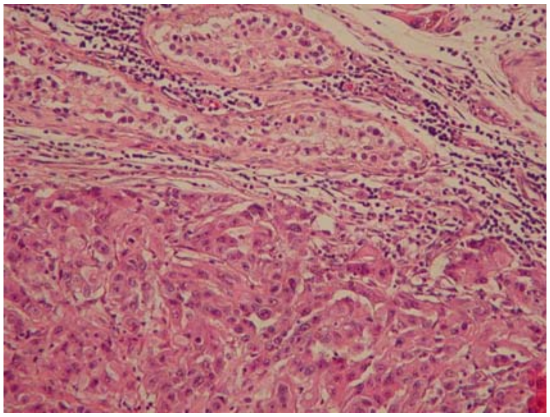

**FIGURE 1.** Tumour amongst normal testis.

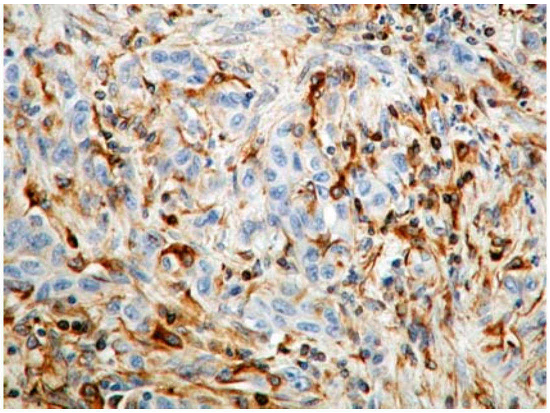

**FIGURE 2.** Vimentin positivity.

**FIGURE 3**. Cytokeratin 7 positivity.
